# Structural Controllability of Temporal Networks with a Single Switching Controller

**DOI:** 10.1371/journal.pone.0170584

**Published:** 2017-01-20

**Authors:** Peng Yao, Bao-Yu Hou, Yu-Jian Pan, Xiang Li

**Affiliations:** 1 Adaptive Networks and Control Lab, Department of Electronic Engineering, Fudan University, Shanghai 200433, China; 2 Research Center of Smart Networks & Systems, School of Information Science & Engineering, Fudan University, Shanghai 200433, China; Beihang University, CHINA

## Abstract

Temporal network, whose topology evolves with time, is an important class of complex networks. Temporal trees of a temporal network describe the necessary edges sustaining the network as well as their active time points. By a switching controller which properly selects its location with time, temporal trees are used to improve the controllability of the network. Therefore, more nodes are controlled within the limited time. Several switching strategies to efficiently select the location of the controller are designed, which are verified with synthetic and empirical temporal networks to achieve better control performance.

## Introduction

Since the seminal work of the Watts-Strogatz (WS) and Barabási-Albert (BA) models [1][2], we have a better understanding of our real world from the perspective of complex network science. With the development of portable electronic devices nowadays, people find that many real-world networks, generated from e-mail contacts [3], instant messages [4], online forums [5] and WiFi records [6–9], contain a plenty of temporal information which yields temporal networks [10]. Compared to static networks, temporal networks have their own characteristics such as bursts and the power-law distributions of contact intervals [11, 12].

Not satisfied with understanding complex networks (no matter they are temporal or not), people are more willing to reform and control complex networks to improve their performances. Therefore, the control of complex networks which has various significant branches such as pinning control [13–20] and model predict control [21, 22], has attracted wide attention over the decades. As applications, network control has been investigated in various areas, such as human brain networks [23] and smart grids [24, 25].

State controllability in control theory [26] describes the ability that a system can traverse from any initial state to any desired state with proper inputs. Structural controllability, which emphasizes the system structure and avoids potential parameter perturbations, was proposed for Linear Time-Invariant (LTI) systems [27]. Poljak transformed the maximum cycle partition problem into an integer linear problem to get the generic dimension of the controllable subspace, and pointed out that the generic dimension of the controllable subspace, which measures the structural controllability of a system, stays the same when its non-zero parameters change [28, 29]. Lombardi and Hörnquist gave a necessary topological condition to network controllability [30]. Based on the maximum matching algorithm, Liu *et al.* located the minimum driver nodes to achieve the structural controllability of a complex network [31].

More efforts were devoted to taming structural controllability of complex networks. In [32], strongly connected components determine the input of a network as well as the leader in a multi-agent system. Ruths *et al.* described the network control profile by defining three control-inducing structures which conceive driver nodes: source, internal-dilation, and external-dilation [33]. Moreover, Jia *et al.* divided nodes into three types (critical, intermittent or redundant) according to their probabilities of being driver nodes [34]. The ability of a single node to control the whole network is denoted as control centrality, and it is determined by node’s hierarchy in the network [35]. Not limited to the static networks whose topologies are fixed, there are works to investigate structural controllability on networked systems with time-varying topologies. In [36], the necessary and sufficient conditions were stated to ensure structural controllability on switched linear systems from the view of graph theory. As for the temporally switched networks and the associated switched systems, the state controllability criterion was obtained in [37], which with the proposed *n*-walk theory, revealed that the *n* temporally independently walks are essential to the structural controllability as well as the strong structural controllability [37]. To measure the ability of a fixed controller, temporal trees of a temporal network were classified to precisely estimate the control centrality of the node [38, 39].

In this article, to improve the structural controllability of a temporal network, a switching controller which selects its location at some specific time points is introduced. When the controller is no longer fixed on a specific node, more diverse temporal trees emerge in the network, which would contribute to improve the network controllability. Several controller switching strategies are proposed to select the controller’s location (i.e. determine the controller’s contact sequence) to achieve high efficiency, which are verified with synthetic and empirical temporal networks.

## Results

In a temporal network, every node and edge might be valid (or activated) only at some specific time points. A temporal network is associated with a Linear Time-Variant (LTV) system as [38]

$$\begin{array}{c} \frac{X(k+1)-X(k)}{t_{k+1}-t_{k}}=A_{k+1}^{T}X\left(k\right)+B_{k+1}U\left(k\right) \end{array}$$ (1)

where *k* ∈ {0, 1, ⋯, *T* − 1}, *t*_*k*+1_ > *t*_*k*_. $X\left(k\right)={\left[x_{1}\left(k\right),x_{2}\left(k\right),\cdots ,x_{N}\left(k\right)\right]}^{T}\in R^{N}$ is the vector of nodes, *N* denotes the number of nodes in the network. $U\left(k\right)={\left[u_{1}\left(k\right),u_{2}\left(k\right),\cdots ,u_{M}\left(k\right)\right]}^{T}\in R^{M}$ is the vector of input signals from controllers outside the network. $A_{k+1}\in R^{N\times N}$ denotes the adjacency matrix of the network at time point *k* + 1, and $A_{k+1}^{T}$ denotes its transpose matrix. Besides, the node set of the network is denoted as *V* = {*v*_1_, *v*_2_, ⋯, *v*_*N*_}. ∀*a*_*k*+1,*ij*_ ∈ *A*_*k*+1_, if there is a directed edge from node *i* to node *j* at time point *k* + 1, *a*_*k*+1,*ij*_ ≠ 0. Otherwise, *a*_*k*+1,*ij*_ = 0. $B_{k+1}\in R^{N\times M}$ denotes the input matrix at time point *k* + 1. *M* is the number of external controllers (usually, *M* ≤ *N*). ∀*b*_*k*+1,*ij*_ ∈ *B*_*k*+1_, if controller *j* connects node *i* at time point *k* + 1, *b*_*k*+1,*ij*_ > 0. Otherwise, *b*_*k*+1,*ij*_ = 0. Pair (*A*_*k*+1_, *B*_*k*+1_) describes a network with its external controllers at time point *k* + 1 as well as its associated system. The discretized temporal network is then described by a sequence of pair (*A*_1_, *B*_1_), (*A*_2_, *B*_2_), ⋯, (*A*_*T*_, *B*_*T*_).

### Temporal network controllability

System $\left(\tilde{A},\tilde{B}\right)$ has the same structure with system (*A*, *B*) if the zero parameters of the matrices are fixed in the same entries. And a system $\left(\tilde{A},\tilde{B}\right)$ is structurally controllable, if there exists a state controllable system (*A*, *B*) with the same structure [27]. Therefore, a temporal network is structurally controllable if its associated LTV system (1) is structurally controllable, i.e. there exists a state controllable LTV system with the same structure [38].

Rewrite Eq (1) into the following form with a single controller

$$\begin{array}{c} \frac{X(k+1)-X(k)}{t_{k+1}-t_{k}}=A_{k+1}^{T}X\left(k\right)+b_{k+1}U\left(k\right) \end{array}$$ (2)

where *B*_*k*+1_ is replaced by *b*_*k*+1_ ($b_{k+1}\in R^{N}$). If the single controller is on node *i* at time point *k* + 1, the value of *i*th row (*b*_*k*+1,*i*_) is non-zero, while the values of other rows equal to zero. Note that the temporal network with a fixed controller in [38] is a special case of Eq (2) when *b*_*k*+1_ is a constant matrix with different *k*.

Denote *D*_*k*+1_ = *t*_*k*+1_ − *t*_*k*_ (*t*_*k*+1_ > *t*_*k*_) as the difference of the two neighbouring time points, $G_{k+1}=I_{k+1}+D_{k+1}A_{k+1}^{T}$, *H*_*k*+1_ = *D*_*k*+1_ *b*_*k*+1_, 0 ≤ *k* ≤ *T* − 1, where *I*_*T*_ = *I*_*T*−1_ = ⋯ = *I*_1_ = *I*, $I\in R^{N\times N}$ is the identity matrix. Note that there is no restriction that *D*_*i*_ = *D*_*j*_ for *i* ≠ *j*, and 1 < *i*, *j* ≤ *T*, so the discrete process doesn’t require periodic sampling.

With Eq (2), *X*(*T*) can be calculated as

$$\begin{array}{ccc} X(T) & = & G_{T}X\left(T-1\right)+H_{T}U\left(T-1\right)\\ & = & G_{T}G_{T-1}X\left(T-2\right)+G_{T}H_{T-1}U\left(T-2\right)+H_{T}U\left(T-1\right)\\ & \cdot & \\ & \cdot & \\ & \cdot & \\ & = & \left[G_{T}\cdots G_{1}\right]\cdot X\left(0\right)+W_{c}\cdot {\left[u(0),u(1),\cdots ,u(T-1)\right]}^{T} \end{array}$$ (3)

where *W*_*c*_ = [*G*_*T*_⋯*G*_2_ *H*_1_, ⋯, *G*_*T*_ *H*_*T*−1_, *H*_*T*_].

Rewriting Eq (3) as

$$\begin{array}{c} W_{c}\cdot {\left[u(0),u(1),\cdots ,u(T-1)\right]}^{T}=X\left(T\right)-\left[G_{T}\cdots G_{1}\right]\cdot X\left(0\right) \end{array}$$ (4)

*rank*(*W*_*c*_) measures the dimension of controllable subspace. When *rank*(*W*_*c*_) = *n* (1 ≤ *n* ≤ *N*), the network is divided into two parts: the part with *n* structurally controllable nodes, and the part with other *N* − *n* nodes.

To quantify the ability of a controller, suppose that *rank*(*W*_*c*_) is the dimension of controllable subspace of the temporal network (*A*_*k*+1_, *b*_*k*+1_), *k* ∈ {0, 1, ⋯, *T* − 1} with node set *V* = {*v*_1_, *v*_2_, ⋯, *v*_*N*_} and a switching controller *I*^*E*^. And a corresponding network is introduced with the dimension of controllable subspace $rank({\hat{W}}_{c})$ and with node set $\hat{V}=\left\{v_{0}\right\}\cup V$. A controller ${\hat{I}}^{E}$ is fixed on node *v*_0_ and *v*_0_ has the same contact sequence (recording nodes it connects at each time point) with the switching controller *I*^*E*^ in the original network.

In (*A*_*k*+1_, *b*_*k*+1_), *A*_*k*+1_ and *b*_*k*+1_ record the topology of internal nodes and the location of the switching controller *I*^*E*^, 0 ≤ *k* ≤ *T* − 1, respectively. Since node *v*_0_ with the fixed controller ${\hat{I}}^{E}$ connects the same nodes as the switching controller *I*^*E*^ does, we have ${\hat{A}}_{k+1}=(\begin{array}{cc} 0 & 0_{1\times N}\\ b_{k+1} & A_{k+1} \end{array})$, and ${\hat{b}}_{k+1}={\left[1,0_{1\times N}\right]}^{T}$, 0 ≤ *k* ≤ *T* − 1. Especially, ${\hat{b}}_{0}={\left[1,0_{1\times N}\right]}^{T}$, ${\hat{A}}_{0}=0_{\left(N+1)\times (N+1\right)}$.

Therefore, ${\hat{H}}_{k}=D_{k}{\hat{b}}_{k}=D_{k}{\left[1,0_{1\times N}\right]}^{T}$, and ${\hat{G}}_{k}={\hat{I}}_{k}+D_{k}{\hat{A}}_{k}=(\begin{array}{cc} 1 & 0_{1\times N}\\ H_{k} & G_{k} \end{array})$, 0 ≤ *k* ≤ *T*,

$$\begin{array}{c} {\hat{W}}_{c}=\left[{\hat{G}}_{T}\cdots {\hat{G}}_{1}{\hat{H}}_{0},{\hat{G}}_{T}\cdots {\hat{G}}_{2}{\hat{H}}_{1},\cdots ,{\hat{G}}_{T}{\hat{H}}_{T-1},{\hat{H}}_{T}\right] \end{array}$$ (5)

where ${\hat{H}}_{T}=D_{T}{\left[1,0_{N}\right]}^{T}$,

${\hat{G}}_{T}{\hat{H}}_{T-1}=D_{T-1}{\left[1,H_{T}\right]}^{T}$,

⋅

⋅

⋅

${\hat{G}}_{T}\cdots {\hat{G}}_{2}{\hat{H}}_{1}=D_{1}{\left[1,H_{T}+G_{T}H_{T-1}+\cdots +G_{T}\cdots G_{3}H_{2}\right]}^{T}$,

${\hat{G}}_{T}\cdots {\hat{G}}_{1}{\hat{H}}_{0}=D_{0}{\left[1,H_{T}+G_{T}H_{T-1}+\cdots +G_{T}\cdots G_{2}H_{1}\right]}^{T}$.

We can easily get the following matrix with the same rank

$\left(\begin{array}{ccccc} 0 & 0 & \cdots & 0 & 1\\ G_{T}\cdots G_{2}H_{1} & G_{T}\cdots G_{3}H_{2} & \cdots & H_{T} & 0_{N\times 1} \end{array})=(\begin{array}{cc} 0_{1\times N} & 1\\ W_{c} & 0_{N\times 1} \end{array}\right)$

Therefore, $rank\left({\hat{W}}_{c}\right)=rank\left(W_{c}\right)+1$. $rank({\hat{W}}_{c})$ measures the ability of the controller. Since the gap between *rank*(*W*_*c*_) and $rank({\hat{W}}_{c})$ is 1, in the following part we also use *rank*(*W*_*c*_) to measure the ability of the controller. To improve the number of the controlled nodes, the controller should select its location properly with the help of temporal trees.

### Temporal trees with a switching controller

To define the temporal trees of a temporal network, we first describe a network in the time-ordered graph (TOG) [40] denoted as *N*(*V*_*T*_, *E*_*T*_). *V*_*T*_ is the node set of TOG including *T* + 1 duplications of both nodes and the external controller, and the edge set *E*_*T*_ contains three types of edges:

1. Edges from node *i*_*k*+1_ to node *j*_*k*+2_ represent the edges from node *i* to node *j* at time point *k* + 1, 0 ≤ *k* ≤ *T* − 1.
2. Edges from node *i*_*k*+1_ to node *i*_*k*+2_ represent that without any outside input from neighbouring nodes or the controller, node *i* transfers its current state to the coming time points.
3. Edges from the external controller $I_{k+1}^{E}$ to node *i*_*k*+2_ represent the edges from controller *I*^*E*^ to node *i* at time point *k* + 1.

**Fig 1:**
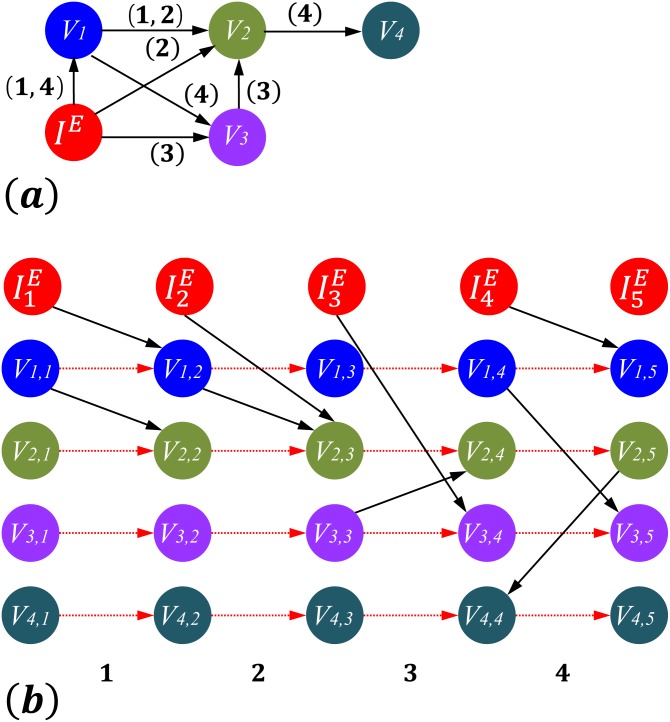
Time-ordered Graph. An example of (a) a temporal network and (b) its corresponding time-ordered graph. The network has 4 nodes {*v*_1_, *v*_2_, *v*_3_, *v*_4_} and an external controller *I*^*E*^. The numbers in parentheses in (a) represent time points 1, 2, 3, 4 when the edges are valid. In (b), each node (including the controller) in (a) has 5 duplications (*v*_1_ in (a) while *v*_1,1_, *v*_1,2_, *v*_1,3_, *v*_1,4_, *v*_1,5_ in (b), etc.). The solid edges are either the edges between nodes, or from controller *I*^*E*^ to a specific node. The dashed edges are from a node to its self-duplication during the neighbouring time points.

Fig 1 shows an example of temporal network and its corresponding time-ordered graph. The network has 4 nodes {*v*_1_, *v*_2_, *v*_3_, *v*_4_} and an external controller *I*^*E*^. Fig 1(a) illustrates the topology of the temporal graph from *k* = 0 to 3. The numbers in parentheses of Fig 1(a) represent the time points when the edges are valid.

In Fig 1(b), each node (including the controller) in Fig 1(a) has 4 + 1 = 5 duplications (*v*_1_ in Fig 1(a) while *v*_1,1_, *v*_1,2_, *v*_1,3_, *v*_1,4_, *v*_1,5_ in Fig 1(b), etc.). The solid edges in Fig 1(b) are either the edges between nodes, or from controller *I*^*E*^ to a specific node. The dashed edges are from a node to its self-duplication during the neighbouring time points. Therefore, both the topology and temporal information remain in the TOG.

In [38], we have proved that the reachability of the fixed controller on the TOG represents the ability of the controller on the origin temporal network. We then generalize the conclusion that the reachability of a switching controller on the TOG also equals the ability of the controller on the origin temporal network.

Define the dynamic communicability matrix [41] as

$$\begin{array}{c} Q=\left(I+aA_{1}\right)\left(I+aA_{2}\right)\cdots \left(I+aA_{T}\right) \end{array}$$ (6)

where *A*_*k*+1_ is the adjacency matrix at time point *k* + 1, 0 ≤ *k* ≤ *T* − 1, and 0 < *a* < 1/*ρ* (*ρ* is the maximum spectral radius of matrices *A*_*k*+1_). Therefore, we have the communicability matrix of controller *I*^*E*^ in a temporal network as

$$\begin{array}{c} Q_{k+1}=\left(I^{*}+a_{k+1}A_{k+1}^{*}\right)\left(I^{*}+a_{k+2}A_{k+2}^{*}\right)\cdots \left(I^{*}+a_{T}A_{T}^{*}\right) \end{array}$$ (7)

where $A_{k+1}^{*}=(\begin{array}{cc} 0 & {\left(b_{k+1}\right)}^{T}\\ 0_{N} & A_{k+1} \end{array})$ denotes the adjacency matrix of the graph at time point *k* + 1 with its external controller *I*^*E*^. The location of the switching controller *I*^*E*^ depends on the non-zero row *j*_*k*+1_ of the input vector *b*_*k*+1_. $I^{*}=(\begin{array}{cc} 0 & 0_{1\times N}\\ 0_{N} & I_{N\times N} \end{array})$. The communicability matrix of controller *I*^*E*^ is used to calculate the reachability of controller *I*^*E*^ at time point *k* + 1, and the reachability from node *i* to node *j* is represented by {*Q*_*k*+1_}_*i*,*j*_. In the case of single switching controller, the *j*_*k*+1_th row of matrix *Q*_*k*+1_ denoted as {*Q*_*k*+1_}_*j*_*k*+1_,∀_ quantifies the reachability of controller *I*^*E*^ on node *j*_*k*+1_ at time point *k* + 1. Selecting the row *j*_1_, *j*_2_, ⋯, *j*_*T*_ from *Q*_1_, *Q*_2_, ⋯, *Q*_*T*_, respectively, we get the reachability matrix of switching controller *I*^*E*^ as:

$$\begin{array}{c} W^{*}=\left[{\left({\left\{Q_{1}\right\}}_{j_{1},\forall }\right)}^{T},{\left({\left\{Q_{2}\right\}}_{j_{2},\forall }\right)}^{T},\cdots ,{\left({\left\{Q_{T}\right\}}_{j_{T},\forall }\right)}^{T}\right] \end{array}$$ (8)

Meanwhile, by the definition of *W*_*c*_, for the *k*th column of matrix *W*_*c*_, *G*_*T*_⋯*G*_*k*_ *H*_*k*−1_ = [(*I* + *A*_*T*_)⋯(*I* + *A*_*k*_)]*H*_*k*−1_. Therefore, the reachability of the switching controller can also be presented by the corresponding row of the communicability matrix. Since the reachability matrix is gathered by the given row of communicability matrices, *rank*(W*) = *rank*(*W*_*c*_).

Using the Breadth-First Search (BFS) in the TOG, we obtain temporal trees *TT*_*k*+1_ from the TOG. Every temporal tree is rooted at the external controller. The input coming from the external controller spreads among nodes via temporal trees.

We define a reachability vector to present the corresponding temporal tree, which starts at time point *k* + 1, as $R_{TT_{k+1}}={\left(0,♯,\cdots ,1,♯,\cdots ,♯\right)}^{T}\in R^{(N+1)\times 1},0\leq k\leq T-1$. If at time point *k* + 1, the controller points at node *j*_*k*+1_, the row *j*_*k*+1_ equals to 1. The first row in *R*_*TT*_*k*+1__ is always zero. The values of the rest rows *i* + 1, *i* ≠ 1, *j*_*k*+1_, 1 ≤ *i* ≤ *N* (denoted by symbol ♯) equal to the product of edges’ weights on the path from node *j*_*k*+1_ to node *i*, or zero, if node *i* is not on the temporal tree *TT*_*k*+1_.

Collecting the reachability vector of each tree into the reachability matrix of temporal trees, it is denoted as $W^{R}=\left[R_{TT_{1}}\;R_{TT_{2}}\;\cdots \;R_{TT_{T}}\right]\in R^{(N+1)\times T}$, $R_{TT_{k+1}}\in R^{(N+1)\times 1},0\leq k\leq T-1$. By the definition of the reachability matrix of temporal trees, we easily obtain *rank*(*W*^*R*^) = *rank*(W*). Therefore, with *rank*(*W*^*R*^) = *rank*(W*) and *rank*(W*) = *rank*(*W*_*c*_), we have *rank*(*W*^*R*^) = *rank*(*W*_*c*_).

**Definition 1** [38] *Temporal trees with the same structure (containing the same nodes and edges) are homogeneously structured trees. Temporal trees with the different structure (containing different nodes or edges) are heterogeneously structured trees*.

In [38], we have proved that the increase number of heterogeneously structured trees would enlarge the controllable subspace of the network (more nodes are controlled). As the external controller *I*^*E*^ switches among different nodes of the network, we assume that controller *I*^*E*^ connects *p* different nodes in all (nodes *P*_1_, *P*_2_, ⋯, *P*_*p*_), 1 ≤ *p* ≤ *N*, during time period 0 ≤ *k* ≤ *T* − 1. The controller connects node *P*_*i*_ ∈ {*P*_1_, *P*_2_, ⋯, *P*_*p*_} at time point *t*_*P*_*i*_,1_, *t*_*P*_*i*_,2_, ⋯, *t*_*P*_*i*_, *h*_*i*__, 1 ≤ *h*_*i*_ ≤ *T* − *p* + 1, respectively. We rewrite *W*^*R*^ as

$$\begin{array}{c} W^{R}=(\begin{array}{cccc} W_{P_{1}}^{R} & W_{P_{2}}^{R} & \cdots & W_{P_{p}}^{R} \end{array}) \end{array}$$ (9)

In the same submatrix $W_{P_{i}}^{R},1\leq i\leq p$, because the controller is on the same node *P*_*i*_, each submatrix $W_{P_{i}}^{R}$ is a collection of temporal trees with a fixed controller.

When *p* = 1, we have $W^{R}=W_{P_{1}}^{R}$, i.e. the external controller *I*^*E*^ is on the same node *P*_1_ as a fixed controller. That is, temporal networks with a fixed controller is a special case of temporal networks with a switching controller. In the case when the controller stays at the same node (temporal trees which are represented by the same submatrix), it will not improve the number of the controlled nodes.

**Theorem 1** *Temporal trees represented by different submatrices* $W_{P_{i}}^{R}$ *are heterogeneously structured trees*.

*Proof:* Take the reachability vectors of temporal trees *R*_*TT*_*k*_*i*___, *R*_*TT*_*k*_*j*___ from $W_{P_{i}}^{R}$ and $W_{P_{j}}^{R}$, *i* ≠ *j*, respectively, and combine them into reachability matrix $W_{2}=(\begin{array}{c} R_{TT_{k_{i}}},R_{TT_{k_{j}}} \end{array})$. Noting that element 1 locates at row *i*, *j*, *i* ≠ *j* (suppose *i* < *j* without loss of generality), we have

$$\begin{array}{c} W_{2}={\left(\begin{array}{cccccccccccc} 0 & ♯ & \cdots & ♯ & 1 & ♯ & \cdots & \cdots & \cdots & \cdots & \cdots & ♯\\ 0 & ♯ & \cdots & \cdots & \cdots & \cdots & \cdots & ♯ & 1 & ♯ & \cdots & ♯ \end{array}\right)}^{T} \end{array}$$ (10)

Similarly, we have the following matrix after linear transforming,

$$\begin{array}{c} {\bar{W}}_{2}={\left(\begin{array}{cccccc} 0 & 1 & ♯ & ♯ & \cdots & ♯\\ 0 & 0 & 1 & ♯ & \cdots & ♯ \end{array}\right)}^{T} \end{array}$$ (11)

in which $rank({\bar{W}}_{2})=2$. It comes to special cases when elements in the same column with 1 are relevant. But even a trivial perturbation would break the relevancy and transform the special case into a normal one. So *TT*_*k*_*i*__ and *TT*_*k*_*j*__ have different structures, and these two temporal trees are heterogeneously structured trees.

Extending *W*_2_ to *W*_3_ by adding reachability vector *R*_*TT*_*k*_*r*___ from $W_{p_{r}}^{R}$, *r* ≠ *i*, *j*, 1 ≤ *r* ≤ *N*. We have $W_{3}=(\begin{array}{c} W_{2},R_{TT_{k_{r}}} \end{array})$. Generally, we have the following matrix

$$\begin{array}{c} {\bar{W}}_{3}={\left(\begin{array}{ccccccc} 0 & 1 & ♯ & ♯ & ♯ & \cdots & ♯\\ 0 & 0 & 1 & ♯ & ♯ & \cdots & ♯\\ 0 & 0 & 0 & 1 & ♯ & \cdots & ♯ \end{array}\right)}^{T} \end{array}$$ (12)

in which $rank({\bar{W}}_{3})=3$. So the trees whose reachability vectors are in *W*_3_ have different structures, and they are heterogeneously structured trees.

Therefore, we extend to *W*_*p*_ that includes *p* reachability vectors, each of which is chosen from different submatrix $W_{i}^{R}$, 1 ≤ *i* ≤ *p* in *W*^*R*^, respectively. Generally, *W*_*p*_ also has a full rank, which means that the trees with different nodes which the controller connects have heterogeneous structures.

With the increase of *p* (the total number of nodes which the controller connects), more heterogeneous trees are generated which may lead to the improvement of ability of the controller.

### Controller switching strategies

Not only the number of nodes which the controller connects but also their reachabillity determine the ability of the controller. Therefore, to get more controlled nodes, effective controller switching strategies to determine the contact sequence are needed.

At first, we obtain the upper bound of the controlled nodes at time point *T*.

**Theorem 2** *By any controller switching strategy, the ability of a controller satisfies that* *rank*(*W*_*c*_) ≤ *min*(*N*, *T*), *where* *N* *is the total number of nodes, and* *T* *is the total time points*.

*Proof:* According to Eq (3),

$$\begin{array}{c} W_{c}=W_{c}\left(T\right)=\left[G_{T}\cdots G_{2}H_{1},\cdots ,G_{T}H_{T-1},H_{T}\right] \end{array}$$ (13)

where $G_{k+1}=I_{k+1}+D_{k+1}A_{k+1}^{T}$, *H*_*k*+1_ = *D*_*k*+1_ *b*_*k*+1_, *D*_*k*+1_ = *t*_*k*+1_ − *t*_*k*_(*t*_*k*+1_ > *t*_*k*_), 0 ≤ *k* ≤ *T* − 1. Since *A*_*k*+1_ is the adjacency matrix of the temporal network at time point *k* + 1, $A_{k+1}\in R^{N\times N}$, 0 ≤ *k* ≤ *T* − 1. Therefore, we have $G_{k+1}=I_{k+1}+D_{k+1}A_{k+1}^{T}\in R^{N\times N}$, and the product of $G_{k+1}\in R^{N\times N}$. Adding a single controller on the network, we have $b_{k+1}\in R^{N}$, so $H_{k+1}=D_{k+1}b_{k+1}\in R^{N}$. For each submatrix *G*_*T*_⋯*G*_*k*+2_ *H*_*k*+1_ in Eq (13), when there is a single controller, $G_{T}\cdots G_{k+2}H_{k+1}\in R^{N}$ is a vector, 0 ≤ *k* ≤ *T* − 1. Therefore, in Eq (13), there are $W_{c}\in R^{N\times T}$, and *rank*(*W*_*c*_) ≤ *min*(*N*, *T*).

Note that *rank*(*W*_*c*_) ≤ *min*(*N*, *T*) represents the upper bound of the controlled nodes. The number of the controlled nodes could never be larger than the total number of network nodes. Meanwhile, during the neighbouring time points, a node can only transport its signal to neighbours, while other nodes are unreachable. Therefore, at time point *k* + 1, 0 ≤ *k* ≤ *T* − 1, we have *rank*(*W*_*c*_(*k* + 1)) = *rank*([*G*_*k*+1_⋯*G*_2_ *H*_1_, ⋯, *G*_*k*+1_ *H*_*k*_, *H*_*k*+1_]) = ≤*min*(*N*, *k* + 1).

We assume that *H*_*k*+1_, 0 ≤ *k* ≤ *T* − 1, is a non-zero vector. *H*_*k*+1_ is a zero vector when controller *I*^*E*^ does not connect any node of at time point *k* + 1. To improve the number of controlled nodes, the assumption that the controller is always activated is reasonable.

**Theorem 3** *At time point* *k* + 1, 0 ≤ *k* ≤ *T* − 1, *if the external controller* *I*^*E*^ *connects to node* *j*_*k*+1_ (*the* *j*_*k*+1_ *th row of* *H*_*k*+1_ *is non-zero), the influence of signal* *u*_*k*_ *from the controller on node* *j*_*k*+1_ *will last till the final time point* *T*.

*Proof:* Since $G_{k+1}=I+D_{k+1}A_{k+1}^{T}$, where ∀[*A*_*k*+1_]_*ij*_ = *a*_*ij*_ ≥ 0 and *D*_*k*+1_ ≥ 0, we have ∀*g*_*ij*_ ∈ *G*_*k*+1_ ≥ 0, and ∀*g*_*ii*_ ∈ *G*_*k*+1_ > 0, 1 ≤ *i*, *j* ≤ *N*, 0 ≤ *k* ≤ *T* − 1. Note that all the elements in *G*_*k*+1_ are nonnegative, especially, its diagonal elements. In the product of several matrices *G*_*k*+1_, all the diagonal elements are also non-zero. We rewrite Eq (3) as

$$\begin{array}{ccc} X(T) & = & (\prod_{k=0}^{T-1}G_{T-k})X\left(0\right)+\\ & & \sum_{k=0}^{T-2}[(\prod_{h=0}^{T-k-2}G_{T-h})H_{k+1}U\left(k\right)]+H_{T}U\left(T-1\right) \end{array}$$ (14)

where the diagonal elements of *G*_*T*_ *G*_*T*−1_⋯*G*_*k*+2_, 0 ≤ *k* ≤ *T* − 2, are non-zero. Since *H*_*k*+1_ is not a zero vector, supposing that its row *j*_*k*+1_ is a non-zero element, we have a non-zero vector *G*_*T*_ *G*_*T*−1_⋯*G*_*k*+2_ *H*_*k*+1_ with its non-zero element at row *j*_*k*+1_. Therefore, when *U*_*k*_ is not a null signal, *G*_*T*_ *G*_*T*−1_⋯*G*_*k*+2_ *H*_*k*+1_ *U*_*k*_ is non-zero. This implies that the influence of *U*(*k*) on node *j*_*k*+1_ will remain at node *j*_*k*+1_ till the last time point *T*. The non-zero *H*_*T*_ *U*(*T* − 1) shows that *U*(*T* − 1) has an influence on a specific node with the controller at the last time point *T*. Hence, with the independency among *x*(0) and *U*(*k*), 0 ≤ *k* ≤ *T* − 1, when input signal *U*(*k*) is on node *j*_*k*+1_ (the row *j*_*k*+1_ of *H*_*k*+1_ is non-zero), it will influence the final state of node *j*_*k*+1_.

As a result, the states of nodes in the network depend not only on their current states and inputs but also on previous states and inputs. This “memory-like” effect within nodes makes them similar to integrators in the continuous-time domain or accumulators in the discrete-time domain. Based on this phenomenon, if the controller selects its location properly, the controlled part remains even if the controller switches to other locations in next time points.

By definition, *W*_*c*_ = [*G*_*T*_⋯*G*_2_ *H*_1_, ⋯, *G*_*T*_ *H*_*T*−1_, *H*_*T*_]. To maximize the ability of the controller, with the knowledge of [*G*_*T*_⋯*G*_2_, ⋯, *G*_*T*_, *I*], we can select proper columns from *G*_*T*_⋯*G*_2_, ⋯, *G*_*T*_, *I*, respectively. However, this controller switching strategy (labeled by “Global” in simulations) requires that we know all the adjacency matrices *A*_*k*+1_, 0 ≤ *k* ≤ *T* − 1 from the start time point 1 to the final time point *T*, which are hard to acquire in an empirical temporal network.

On the other hand, for most nodes in a temporal network, nodes with large aggregated degree tend to have the higher control centrality [38]. In fact, the aggregated degree of a node represents the total number of its neighbours during the whole time period, which implies its ability to broadcast the signal in the network. Therefore, we design two controller switching strategies as follows. We first shuffle the node sequence 1, 2, ⋯, *N* as a contact sequence where the nodes with large aggregated degrees have a priority to be connected by the controller (labeled by “Descend”). To make a comparison, we also shuffle the node sequence 1, 2, ⋯, *N* as a contact sequence where the nodes with small aggregated degrees have a priority to be connected by the controller (labeled by “Ascend”).

The above three controller switching strategies require global topology information of the temporal network, such as adjacency matrix at every time point or aggregated degree over the whole time period. Based on currently available topology information, we propose a controller switching strategy labeled by “Greedy”. By the greedy algorithm, at time point *k* + 1, we get *H*_*k*+1_ that maximize the rank of [*G*_*k*+1_ *G*_*k*_⋯*G*_2_ *H*_1_, *G*_*k*+1_ *G*_*k*_⋯*G*_3_ *H*_2_, ⋯, *H*_*k*+1_]. As a result, we get the local optimum solution at the current time point. In this way, from time point 1 to *T*, we choose *H*_1_, *H*_2_, ⋯, *H*_*T*_, respectively. Therefore, we get the whole contact sequence of the switching controller. In this case, at time point *k* + 1, what we need is *A*_1_, *A*_2_, ⋯, *A*_*k*+1_, while the “future” topology information (such as *A*_*k*+2_, *A*_*k*+3_, ⋯, *A*_*T*_) is not required.

### Numerical simulations

#### Synthetic networks

**Fig 2:**
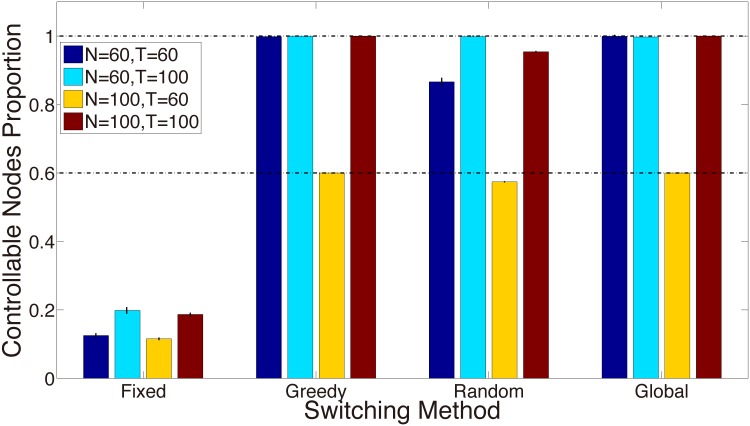
Controller switching strategies in synthetic networks. The histogram labeled by ‘Fixed’ shows the normalized number of the controlled nodes when the controller is fixed on every node in the network. The histogram labeled by ‘Greedy’ shows the normalized number of the controlled nodes when determining the contact sequence of the controller only requires the past and current topology information. The histogram labeled by ‘Random’ shows the normalized number of the controlled nodes when the controller switches among nodes randomly. The histogram labeled by ‘Global’ shows the normalized number of the controlled nodes when determining the contact sequence of the controller requires the topology information during the whole time period. The vertical bars represent the standard deviations. The ER networks with 60 and 100 nodes are generated. The edge generating probability is *P* = 0.002. At each time point *k* + 1, 0 ≤ *k* ≤ *T* − 1, *T* = 60 and 100, we generate the ER networks independently.

We define that the controller switches its location every *l* time points, i.e. the input matrices *b*_*k*_ = *b*_*k*+1_ = ⋯ = *b*_*k*+*l*_, *k* = 1, 1 + *l*, 1 + 2*l*, ⋯, *k* ≤ *T*. At first, we set *l* = 1, which means that the controller can switch its location at every time point. In Fig 2, three controller switching strategies (“Global”, “Greedy” and “Random”) as well as the “Fixed” strategy are computed with the ER temporal networks with the total node number *N* = 60 and 100, and the final time point *T* = 60 and 100, respectively. The result of the “Fixed” strategy is the maximum number of the controlled nodes when the controller is fixed on every node in the network.

After normalizing the number of the controlled nodes (i.e. *rank*(*W*_*c*_)/*N*), as shown in Fig 2, all the controller switching strategies lead to the increase of the number of the controlled nodes over the fixed strategy. By using the topology information of adjacency matrices, the “Global” strategy and the “Greedy” strategy improve controllability more than the “Random” strategy does, especially when *T*/*l* is numerically close to *N* (e.g., *N* = 100, *T* = 100). From Fig 2, with sufficient time points, i.e. *T*/*l* ≥ *N*, the normalized number of the controlled nodes with the switching controller can arrive at 1, i.e. the whole network is structurally controllable. Otherwise, in the network of *N* > *T*/*l* (e.g., *N* = 100, *T* = 60) in Fig 2, the normalized number of the controlled nodes of the controller can’t be larger than *T*/(*lN*).

**Fig 3:**
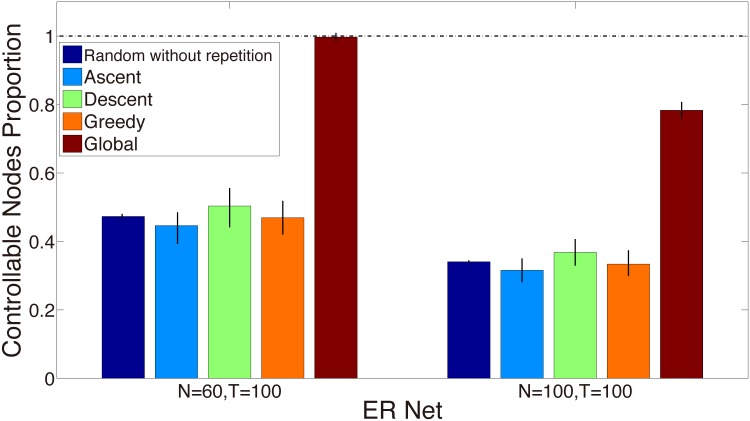
Switching every 5 time points. The histogram labeled by ‘Random without repetition’ shows the number of the controlled nodes when the controller connects nodes randomly without repetition. The histogram labeled by ‘Descend’ shows the number of the controlled nodes when nodes with large aggregated degrees have a priority to be connected by the controller. The histogram labeled by ‘Ascend’ shows the number of the controlled nodes when nodes with less aggregated degrees have a priority to be connected by the controller. The histogram labeled by ‘Greedy’ shows the number of the controlled nodes when determining the contact sequence of the controller using the past and current topology information of *A*_*i*+1_, 0 ≤ *i* ≤ *k*, 0 ≤ *k* ≤ *T* − 1, *T* = 100. The histogram labeled by ‘Global’ shows the number of the controlled nodes when determining the contact sequence of the controller using the global topology information of *A*_*k*+1_, 0 ≤ *k* ≤ *T* − 1. The ER networks with 60 and 100 nodes are generated. The vertical bars represent the standard deviations. Edges are generated with the probability *P* = 0.002. At each time point *k* + 1, 0 ≤ *k* ≤ *T* − 1, we generate the ER networks independently.

To further investigate the effectiveness of these strategies (“Global”, “Greedy”, “Descend”, “Ascend” and “Random”), we decreasse the switching frequency (set *l* > 1). Therefore, during a limited time period, the controller has fewer times of chance to select proper nodes, which means that it has to make the decision more wisely and efficiently. As shown in Fig 3, the controller changes its location every *l* = 5 time points. Therefore, during the whole time period, the controller has only *T*/*l* = 100/5 = 20 times of opportunities to switch its location. There are two temporal networks in Fig 3, one of which has *N*_1_ = 60 nodes while the other *N*_2_ = 100 nodes, both with the final time point *T* = 100. Since *N*_2_ > *N*_1_ > *T*/*l*, the number of the controlled nodes is mainly limited by the total time points. As a result, all the controller switching strategies in the former network (i.e. *N* = 60, *T* = 100) lead to the large number of the controlled nodes.

In both networks, the “Global” strategy using global topology information *A*_*k*+1_, 0 ≤ *k* ≤ *T* − 1, improves the number of the controlled nodes most significantly, and it is less disturbed by the time limitation compared with other controller switching strategies. On the other hand, shuffling the node sequence 1, 2, ⋯, *N* randomly, labeled as “Random without repetition”, has almost the same result with that of “Greedy” strategy. The “Descent” strategy in which the nodes with higher aggregated degrees are prior connected by the controller leads to a bit of improvement compared with selecting randomly or greedily. In contrast, when the nodes with the lower aggregated degrees are prior connected by the controller in “Ascent” strategy, the controller has the fewest controlled nodes.

#### Empirical network

Hypertext 2009 dynamic contact network (shorted as “HT09”) is an empirical network generated from a dataset collected during a conference [42]. In the conference, the face-to-face proximities of voluntary attendees are recorded as a sequence of contacts. When considering structural controllability on social networks like HT09, we simulate the process that people’s opinion is affected by a leader via their social interactions.

Therefore, we generate a temporal network from the attendees of HT09 and their interactions. In the network, nodes represent attendees, and node states represent their opinions. Edges represent their face-to-face interactions, which are not always active during the whole conference. Since we assume that a leader (controller) wants to find an effective way to affect more attendees’ opinion, the leader can talk/interact with any attendees following some rules, or simply stick to one person all the time.

**Fig 4:**
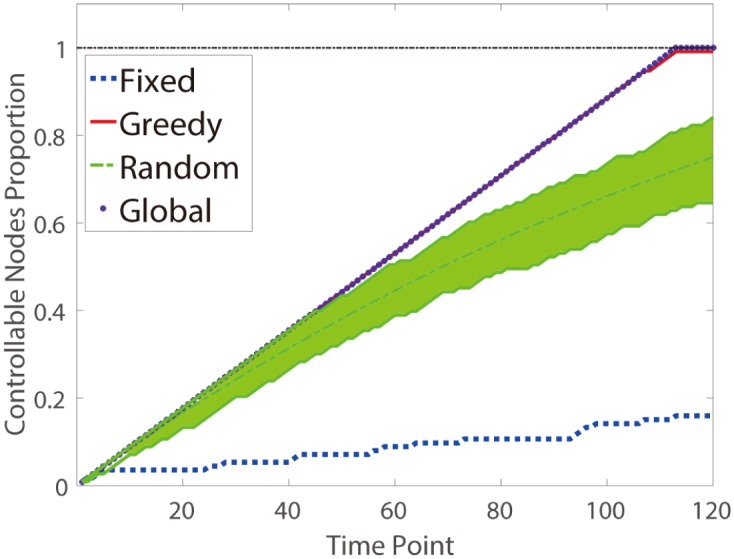
Controller switching strategies in HT09. Temporal network HT09 is a social network with 113 nodes and 120 time points. The curve labeled by ‘Fixed’ shows the number of the controlled nodes when the controller is fixed on the node which has the largest number of the controlled nodes in the network. The curve labeled by ‘Greedy’ shows the number of the controlled nodes when determining the contact sequence of the controller using the past and current topology information of *A*_*i*+1_, 0 ≤ *i* ≤ *k*, 0 ≤ *k* ≤ *T* − 1, *T* = 120. The curve labeled by ‘Random’ shows the mean number of the controlled nodes when the controller switches among nodes randomly. And the lime area represents the range of simulation results by the “random” strategy. The curve labeled by ‘Global’ shows the number of the controlled nodes when determining the contact sequence of the controller using the global topology information of *A*_*k*+1_, 0 ≤ *k* ≤ *T* − 1, *T* = 120.

The controller determines its contact sequence by four strategies including three controller switching strategies (“Global”, “Greedy” and “Random”). At each time point from 1 to 120, the number of the controlled nodes is calculated and plotted in Fig 4. As shown in Fig 4, all the three controller switching strategies contribute to significantly promoting the controllability, compared with the fixed strategy. Among the controller switching strategies, the curves of the “Global” and the “Greedy” strategies almost coincide with each other. However, the number of the controlled nodes by the “Global” strategy converges to the maximum number of the controlled nodes (i.e. *rank*(*W*_*c*_) = *min*(*N*, *T*) = 113), while the number of the controlled nodes by the “Greedy” strategy converges to a local maximum number of the controlled nodes (*rank*(*W*_*c*_) = 112 < *min*(*N*, *T*)). The “Random” strategy makes the poorer improvement than the previous two, but it is still much better than the “Fixed” strategy. As a result, we can see that the leader simply randomly interacting with attendees is an effective way to affect people’s opinion compared with sticking to any popular one. And if the leader could get the knowledge of the whole interactions during the conference, the leader will make the best decision.

## Discussion

In summary, we have transformed a temporal network with a switching controller into the time-ordered graph (TOG) as well as its temporal trees. The location of the controller leads to heterogeneous trees so more nodes in the network could be controlled. As a result, the single switching controller almost makes all the network controlled with sufficient time. While a fixed controller could only control parts of the networks. Four controller switching strategies have been proposed to select the location of the controller. The “Global” strategy requires the knowledge of adjacency matrices over the whole time period. The “Descend” and “Ascend” strategies need the information of the aggregated degrees. The “Greedy” strategy only has to know the adjacency matrices of the past time points. These strategies are verified with both synthetic networks and empirical networks. In general, controller switching strategies that use more temporal topology information gain the higher efficiency. With the “Global” strategy, more nodes are controlled in limited time points. However, since the “Global” strategy (and the “Greedy” strategy as well) selects columns from matrices *G*_*k*_, *k* = 1, 2, ⋯, *T*, to enlarge the rank of *W*_*c*_, this leads to more computational cost and time to find the controller’s proper location, which would be difficult to satisfy when the network size increases. Therefore, we have to make a tradeoff between the demand of computational resource and the strategy performance.

## Methods

### Introduction of controller switching strategies

We have introduced four controller switching strategies to let the controller choose its location in a temporal network.

“Global” strategy: with the knowledge of topologies over the whole time period *G*_*T*_, *G*_*T*−1_, ⋯, *G*_2_, select columns from *G*_*T*_⋯*G*_2_, ⋯, *G*_*T*_, *I* that maximizes *rank*(*W*_*c*_), respectively.

“Greedy” strategy: at each time point *k* + 1, select *H*_*k*+1_ that maximizes the rank of [*G*_*k*+1_ *G*_*k*_⋯*G*_2_ *H*_1_, *G*_*k*+1_ *G*_*k*_ ⋯ *G*_3_ *H*_2_, ⋯, *H*_*k*+1_].

“Descend” strategy: shuffle the node sequence 1, 2, ⋯, *N* as a contact sequence where nodes with large aggregated degrees have a priority to be connected by the controller.

“Ascend” strategy: shuffle the node sequence 1, 2, ⋯, *N* as a contact sequence where nodes with small aggregated degrees have a priority to be connected by the controller.

### Generation of temporal networks

Structural controllability of synthetic and empirical networks are illustrated with two examples in the following part, where edge weights are randomly initialized, and the numerical results are averaged over 500 rounds of realizations.

We generate a synthetic network whose directed edges are activated with probability *P* based on the ER model [43]. Setting *P* as 0.002, we generate the ER networks with *N* (*N* = 60 and 100) nodes at each time point *k* + 1 (0 ≤ *k* ≤ *T* − 1, *T* = 60 and 100), respectively. For simplicity, we assume that *D*_*k*+1_ = *t*_*k*+1_ − *t*_*k*_ = 1.

The records of HT09 are sorted by time, and we collect every 20 neighbouring contact records as 20 temporal edges in a time point *k* + 1, 0 ≤ *k* ≤ 119. Therefore, a temporal network from HT09 yields a temporal network with 113 nodes and 120 time points. At each time point *k* + 1, there are 20 non-zero parameters in *A*_*k*+1_ which represent that 20 edges are valid.
